# Ten simple rules for implementing open and reproducible research practices after attending a training course

**DOI:** 10.1371/journal.pcbi.1010750

**Published:** 2023-01-05

**Authors:** Verena Heise, Constance Holman, Hung Lo, Ekaterini Maria Lyras, Mark Christopher Adkins, Maria Raisa Jessica Aquino, Konstantinos I. Bougioukas, Katherine O. Bray, Martyna Gajos, Xuanzong Guo, Corinna Hartling, Rodrigo Huerta-Gutierrez, Miroslava Jindrová, Joanne P. M. Kenney, Adrianna P. Kępińska, Laura Kneller, Elena Lopez-Rodriguez, Felix Mühlensiepen, Angela Richards, Gareth Richards, Maximilian Siebert, James A. Smith, Natalie Smith, Nicolai Stransky, Sirpa Tarvainen, Daniela Sofia Valdes, Kayleigh L. Warrington, Nina-Maria Wilpert, Disa Witkowska, Mirela Zaneva, Jeanette Zanker, Tracey L. Weissgerber

**Affiliations:** 1 Freelance Open Science researcher, Gladbeck, Germany; 2 QUEST Center for Responsible Research, Berlin Institute of Health at Charité–Universitätsmedizin Berlin, Berlin, Germany; 3 Einstein Center for Neurosciences Berlin, Charité–Universitätsmedizin Berlin, corporate member of Freie Universität Berlin and Humboldt-Universität zu Berlin, Berlin, Germany; 4 Neuroscience Research Center (NWFZ), Charité–Universitätsmedizin Berlin, corporate member of Freie Universität Berlin and Humboldt-Universität zu Berlin, Berlin, Germany; 5 Charité–Universitätsmedizin Berlin, corporate member of Freie Universität Berlin and Humboldt-Universität zu Berlin, Berlin, Germany; 6 Department of Psychology, York University, Toronto, Canada; 7 Population Health Sciences Institute, Faculty of Medical Sciences, Newcastle University, Newcastle Upon Tyne, United Kingdom; 8 Department of Hygiene, Social-Preventive Medicine & Medical Statistics, School of Medicine, Faculty of Health Sciences, Aristotle University of Thessaloniki, University Campus, Thessaloniki, Greece; 9 Melbourne Neuropsychiatry Centre (MNC), Department of Psychiatry, The University of Melbourne & Melbourne Health, Melbourne, Australia, and Melbourne School of Psychological Sciences, University of Melbourne, Melbourne, Australia; 10 Department of Mathematics and Computer Science, Freie Universität Berlin, Berlin, Germany; 11 Department of Psychiatry and Psychotherapy, Charité–Universitätsmedizin Berlin, Berlin, Germany; 12 Institute of Public Health, Charité—Universitätsmedizin Berlin, Berlin, Germany; 13 Department of Psychosomatic Medicine and Psychotherapy, Central Institute of Mental Health Mannheim, Medical Faculty Mannheim / Heidelberg University, Mannheim, Germany; 14 School of Psychology, Dublin City University, Dublin, Ireland; 15 Department of Psychiatry, Icahn School of Medicine at Mount Sinai, New York, New York, United States of America; 16 Seaver Autism Center for Research and Treatment, Icahn School of Medicine at Mount Sinai, New York, New York, United States of America; 17 Department of Genetics and Genomic Sciences, Icahn School of Medicine at Mount Sinai, New York, New York, United States of America; 18 Division of Pulmonary Inflammation, Charité–Universitätsmedizin Berlin, Berlin, Germany; 19 Institute of Functional Anatomy, Charité Universitätsmedizin Berlin, Berlin, Germany; 20 Center for Health Services Research, Brandenburg Medical School Theodor Fontane, Faculty of Health Sciences Brandenburg, Ruedersdorf, Germany; 21 Imperial College, London, United Kingdom; 22 School of Psychology, Faculty of Medical Sciences, Newcastle University, Newcastle upon Tyne, United Kingdom; 23 Meta-Research Innovation Center at Stanford (METRICS), Stanford University, Stanford, California, United States of America; 24 Botnar Research Centre and Centre for Statistics in Medicine, Nuffield Department of Orthopaedics, Rheumatology and Musculoskeletal Sciences, University of Oxford, Oxford, United Kingdom; 25 Department of Psychology, School of Education, Language and Psychology, York St John University, New York, United Kingdom; 26 Department of Pharmacology, Toxicology and Clinical Pharmacy, Institute of Pharmacy, University of Tübingen, Tübingen, Germany; 27 Department of Psychology and Logopedics, Faculty of Medicine, University of Helsinki, Helsinki, Finland; 28 Max Delbrück Center for Molecular Medicine in the Helmholtz Association (MDC), Berlin, Germany; 29 Experimental and Clinical Research Center Charité–Universitätsmedizin Berlin, Berlin, Germany; 30 School of Psychology, Nottingham Trent University, Nottingham, United Kingdom; 31 Department of Neuropediatrics, Charité–Universitätsmedizin Berlin, Berlin, Germany; 32 Division of Psychology and Language Sciences, University College London, London, United Kingdom; 33 Department of Experimental Psychology, University of Oxford, Oxford, United Kingdom; Dassault Systemes BIOVIA, UNITED STATES

## Abstract

Open, reproducible, and replicable research practices are a fundamental part of science. Training is often organized on a grassroots level, offered by early career researchers, for early career researchers. Buffet style courses that cover many topics can inspire participants to try new things; however, they can also be overwhelming. Participants who want to implement new practices may not know where to start once they return to their research team. We describe ten simple rules to guide participants of relevant training courses in implementing robust research practices in their own projects, once they return to their research group. This includes (1) prioritizing and planning which practices to implement, which involves obtaining support and convincing others involved in the research project of the added value of implementing new practices; (2) managing problems that arise during implementation; and (3) making reproducible research and open science practices an integral part of a future research career. We also outline strategies that course organizers can use to prepare participants for implementation and support them during this process.

## Introduction

Open, reproducible and replicable research practices (defined in **Box 1**) are a fundamental part of science, yet training in these skills is not yet typically included in undergraduate or graduate curricula [1]. Training is often organized on a grassroots level, offered by early career researchers (ECRs) for ECRs. These courses can attract students from many fields; hence, sessions typically provide an overview of theoretical concepts and technical tools. While buffet style courses that cover many topics can inspire participants to try new things, they can also be overwhelming. Participants may not know where to start once they return to their research team. Courses rarely focus on how to *implement* practices within a specific research area or project within a research group.

**Box 1. Definitions of Open Science, reproducibility and replicability**

There is no fixed definition of the terms open, reproducible and replicable in the literature, and different disciplines may have different understandings of these terms. The following definitions are used throughout this paper:

**Reproducible:** A research result that can be recreated by others using the same data and analysis pipeline [2].

**Replicable:** A research result that can be recreated by others using independent data and an independent analysis pipeline [2].

**Open Science:** Making the research process and its outputs accessible to everyone. This covers practices such as open access publishing of manuscripts, methods, data and code, open educational resources, and open engagement of societal actors [3].

**Robust research:** Combining Open Science practices and good scientific practice to produce reproducible and replicable research results.

Researchers, especially ECRs, often require support from collaborators and approval from their supervisors to implement practices. Challenges that course participants face when attempting to integrate practices that they have learned in courses into their own research include convincing colleagues and supervisors that changes are needed, deciding which practices to implement, and obtaining additional skills and resources for implementation [4]. Courses rarely discuss strategies for convincing other members of the research team who are involved in the project to implement new practices, or techniques for managing problems that arise during implementation. Resources to guide researchers through this socially and technically complex transition phase, where education meets reality, are lacking.

**Fig 1:**
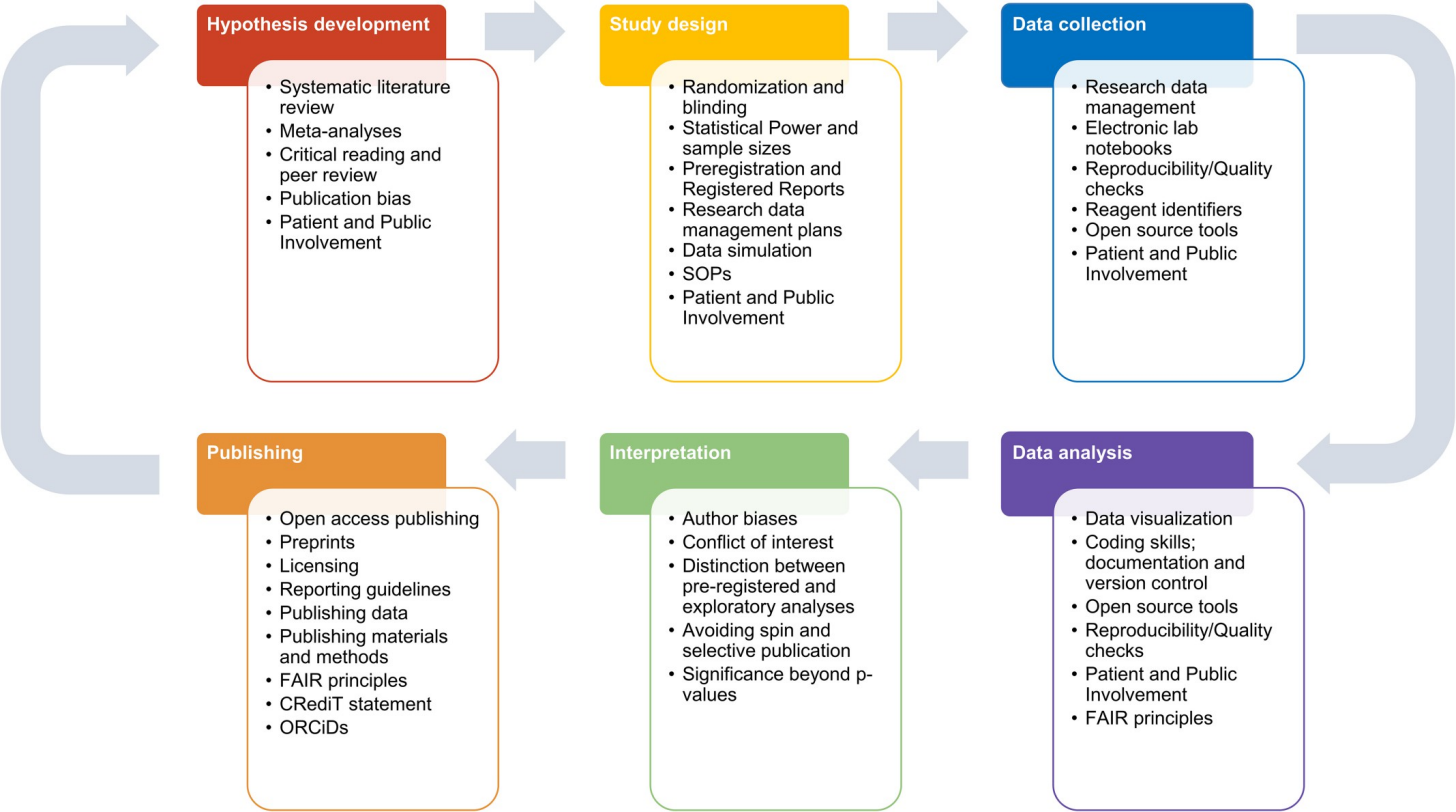
Examples of practices that may be taught in robust research courses. This schematic illustrates concepts and skills taught in our courses and others [7,8], assigned to different phases of the research cycle. This list is not exhaustive and will vary across fields. Some practices are addressed at multiple phases of a project. Abbreviations: CRediT, Contributor Roles Taxonomy; FAIR, findability, accessibility, interoperability, and reuse of digital assets; ORCiD, Open Researcher and Contributor ID; RRID, Research Resource Identifier; SOP, standard operating procedure.

In September 2021, we conducted a virtual brainstorming event with participants of two different five-day robust research courses to find out what transpired after their training. This event format has been described previously [5,6] and is also discussed in the Supporting information (**S1 Text**). The courses that event participants had completed were run between 2018 and 2021 and mostly aimed at biomedical researchers [7,8] (see **Fig 1** for course content).

Previous work has extensively described practices that researchers can adopt to make their own work more robust (e.g., [4,9]). Here, rather than focusing on the practices themselves, we have distilled conversations during the event into ten simple rules to guide participants of relevant training courses in implementing robust research practices in their own projects, once they return to their research group (see **Table 1**). This includes (1) prioritizing and planning which practices to implement, which includes obtaining support and convincing others involved in the research project of the added value that new practices can bring to the research project; (2) managing problems that arise during implementation; and (3) making reproducible research and open science practices an integral part of a future research career. Strategies that course organizers can use to prepare and support participants during this process are described in the Supporting information (**S2 Text**).

**Table 1:** Phases of implementing practices after a robust research course. These 10 simple rules can be organized into three phases of the implementation process. Some steps are relevant at all phases. Scientists may need to revisit some phases or adapt their plans as they encounter barriers and challenges, or decide to implement additional robust research practices in their studies.

Rules	Stages		
	Plan	Implement	Look to the future
Rule 1: Make a shortlist	x		
Rule 2: Join a community	x	x	x
Rule 3: Talk to your study team	x		
Rule 4: Address resistance constructively	x	x	
Rule 5: Decide what to implement. Make a plan.	x	x	
Rule 6: Compromise and be patient		x	
Rule 7: Reassess and adapt your plan		x	
Rule 8: Share best practices and lessons learned			x
Rule 9: Get credit. Make your contributions visible.			x
Rule 10: Seek supportive future employers			x

Some of these rules are also relevant to researchers who learn about reproducible and open science practice through self-study or other means. An important difference between these scenarios is that courses often cover many topics within a short period of time, whereas scientists who learn new practices through self-study can focus on a single practice and move at their own pace.

## Rule 1: Shortlist possible practices to implement in your current project

Robust research courses often cover many practices, and it may be difficult to know where to start when you return to your own research environment. Few research groups have the time and resources to implement everything within a short period of time. Implementing one to three new practices per project is a more feasible approach that allows you to expand your skills over time, while sharing what you have learned with others in your team. Start by creating a shortlist of a few things that you might want to implement in your current project. Be aware that there may be dependencies that affect the order in which you implement practices. Sharing code, for example, might require ensuring that code has been well documented with good version control. A shortlist will help you to prepare for a conversation with members of your research group (**Rule 3**), while keeping your workload and stress levels manageable (**Rule 7**).

When shortlisting robust research practices to implement, focus on practices that are relevant to your field or project and that you can address at your current project stage. Find out whether there are institutional or funder policies that apply to your current project, such as open access or open data policies, or policies related to scientific rigor and integrity. Be mindful of ethical guidelines that may affect the implementation of specific practices, such as open data. Think about what resources you might need to implement various practices, including skills, infrastructure, and support from your colleagues. Talking to your team (**Rule 3**) will help you to clarify whether you have the resources necessary to implement specific practices. Finally, think about your own interests and skills. What practices would be most useful to you in this project and for future career development?

You may want to find or compile a list of “best practice” materials such as articles, reporting guidelines, or online tutorials for items on your shortlist to help with the next steps. Course instructors may have provided this information during your course, and members of your robust research community (**Rule 2**) may also have suggestions.

## Rule 2: Join a community to access expertise and support

Being the only person in your research group who is interested in robust research practices can be daunting. Therefore, it is critical to seek out a robust research community. This may start with the “micro-community” of people in your direct research environment, who are essential for implementing practices in your own projects (see also **Rules 3 to 8**). You may also find allies or collaborators beyond your own research group and department, including people in supporting roles (e.g., libraries, IT services, open science offices), in journal clubs, scientific societies, or other stakeholders (e.g., funders, publishers, industry). Look for materials or talks that they have shared online, or ask them about their experiences in undertaking robust research, including lessons learned and challenges faced.

Joining a “macro-community,” which supports robust research on a broader scale (local, national, or international), is a great way to find additional support and training. Examples of macro-communities are journal clubs (e.g., ReproducibiliTea [10]; https://reproducibilitea.org/) or the international Reproducibility Networks (https://www.ukrn.org/international-networks/). Look for new courses or seminars within your university and other organizations to develop your skills further. These might include skills such as how to communicate change, how to deal with institutional politics, or how to cope with psychosocial aspects associated with implementing change [11,12] (also see **Rule 7**).

## Rule 3: Talk to your research team about what to implement

Consider whose support you might need to implement the practices on your shortlist. Researchers who are working on fairly independent projects within their research team may be able to simply change their own practices, although supervisor approval may be required. Researchers who work on collaborative projects will often need support from collaborators. Support from others in the team will be needed to implement practices that would alter project timelines, require additional resources or necessitate adjustments to standard operating procedures (SOPs).

Once you’ve identified whose support you might need, find out how these people feel about implementing robust research practices. Speak with your supervisor and other team members soon after the course. This could be either in one-on-one conversations or in a group meeting. Prepare for the conversation, for example, with slides describing what you have learned, the shortlist of practices that you could incorporate into your project, and why you selected these practices. Cite evidence, for example, from meta-research (science of science) studies and experiences of others in the scientific community, to show that the practices you would like to implement are beneficial for your team’s research and individual careers.

Explain what you have found useful and important and why. Keep it simple and practical, and try to anticipate concerns that your colleagues might have. Your team will need to think critically when deciding what to implement, so mention any concerns that you may have. Focus on how proposed practices will benefit the research team, but be honest about costs and obstacles. It is important to approach others with a positive attitude. Your goal is not to criticize anyone’s past work, but to suggest future improvements. Scientific practice evolves over time, and other researchers may not want to engage in conversations if they feel their previous work is being dismissed.

When talking to your research group, consider factors outside of your direct research environment that could affect attitudes and decision-making. For example, your institution, funder, or target journal may have policies requiring some of the practices that you propose to implement. Use these policies to show how being ahead of the curve will make manuscript submissions easier and strengthen future grant applications. Your institution may also offer support, training, or tools for implementation, demonstrating that they encourage the practices that you want to implement.

Success stories from the literature or your own experience are powerful tools for convincing others. You might consider arranging a seminar, potentially with speakers who are senior scientists with expertise in the practices that you would like to implement. These speakers can share their experiences and success stories, explain the benefits of certain practices, and offer lessons learned for successful implementation.

## Rule 4: Prepare for resistance and address it constructively

If your research team is fully on board, congratulations! If not, consider why they may not immediately be convinced. They may have concerns that implementing robust research practices can cost time and resources. Assume positive intent. Your supervisor may genuinely be concerned that implementing the practices you propose could take time away from your project or prevent you from achieving your career goals in the current academic system. However, there are many good reasons for everyone to implement robust research practices, including both pull- and push-forces [13]. These reasons may be “selfish” (e.g., increased efficiency or error prevention [14]), motivated by policies at your institution or funder, or linked to improving the transparency, accountability, and reliability of research evidence. Different reasons may resonate with different individuals. Choose arguments that directly address your colleagues’ concerns. Remember that interpersonal factors, including your relationship with your colleagues, body language, and tone, affect how individuals might receive your ideas and whether they support or resist them [11,12,13].

Identify which practices you can implement on your own and which practices require the support of others (e.g., your supervisor). Supportive senior group members can help you to navigate discussions with other team members and supervisors who have lingering concerns.

If others in your research team are highly resistant to implementing practices, you may need to choose your battles. Focus on easy wins (**Rule 5**) and use them as a basis for more complicated discussions later. It is OK if you cannot convince everyone. Take advantage of opportunities to move forward by collaborating with allies in your research group or wider community.

## Rule 5: Decide what to implement and create an implementation plan

Factor in the conversations with your research team to decide which items from your shortlist you would like to implement. Make sure that the practices that you choose to implement are feasible given the time and resources available to you. If you are an ECR, support from your supervisor for implementing practices is very valuable. If the practices that you have selected require others on your research team to make changes, support from these individuals is also particularly important. In many cases, there is no “right” order to implement robust research practices: for example, something that is described as a “low-hanging fruit” in a course might be very challenging depending on your skills, your research topic, and your research environment. Begin with practices that are broadly supported by team members and would be easy for you to implement.

Prepare an implementation plan to guide yourself and others in your research team through the “who,” “what,” “when,” and “how” for each practice that you plan to implement. Try to anticipate challenges that you may encounter during the implementation process. Where can you look for advice or support? You may want to solicit help from people who have already accomplished what you would like to achieve, for example, from your course or community (**Rule 2**). Be flexible with your plan. Things that you learn while implementing one practice might open up unexpected avenues for implementing other practices that you had not considered.

Create a plan for developing additional skills needed to implement the practices that you selected. Learning about robust research practices makes you employable, both inside and outside of academia [15]. Skills such as coding and project management will be valuable for many jobs. Your future employer might appreciate an employee who is proactively involved in changing the status quo and who can develop creative solutions. You might also want to think about more formally developing change management competencies [16] in addition to “learning by doing.” The process of implementing practices will help you to develop transferable skills, including flexibility and adaptability, disagreeing constructively, problem solving, navigating difficult discussions, and providing hands-on training. If your institution or funder uses Individual Development Plans (IDPs), integrate training in robust research practices into your IDP. This will allow you to allocate time and resources towards skill development (e.g., course fees, infrastructure needs for implementing practices). If you are applying for a career development grant, include training in robust practices in your training plan and illustrate how this training will enhance your research project.

## Rule 6: Compromise and be patient

Research improvement is a marathon, not a sprint. Your supervisor or research team might not want to introduce all the practices that you suggest. Be positive and invest time and effort in practices that you can implement independently or have collaboratively agreed to implement. Research practices are evolving constantly, so your colleagues might be more exposed to robust practices over time while reading the literature, going to conferences, or observing the effects of practices that you have already implemented. A patient approach may allow you to introduce more practices in the future.

Compromise is important when implementing practices. Look for opportunities to move towards more robust practices while addressing your collaborators’ reservations. Find out what compromises your collaborators would accept. There may also be legitimate reasons for not being able to implement certain practices (e.g., legal restrictions related to participant data sharing). In these cases, explain why a given practice could not be implemented when drafting the manuscript.

If you have the capacity and feel very strongly about a certain practice, offer to do the work on behalf of other team members. This way, they do not need to invest time in something they are not convinced is useful. However, be mindful of your own workload and personal limits (see **Rule 7**). No matter what you decide to do, be patient with yourself. Implementing everything at once is a huge task, so remember that small changes will add up over the years.

## Rule 7: Reassess and adapt your implementation plan when circumstances change. Look after yourself.

Implementing new research practices can be challenging. Your mental health and well-being are incredibly important. Balance what you want to do with what you can do; do not commit to more than you can achieve. This is especially important in environments with high resistance. Do not waste time arguing with colleagues who are not receptive.

If you feel overwhelmed or lose your motivation, consider reaching out to your networks to look for support and for troubleshooting advice (**Rule 2**). Get a fresh perspective by finding a mentor or buddy who is not a member of your research group, department, or institution. Do not be afraid to say no or take a break if you feel overwhelmed.

Circumstances can change over the life span of a research project, and you may need to reassess your implementation plan. This may include adjusting your expectations or taking a step back based on changes in personal circumstances or unanticipated obstacles. Your priorities might shift, and implementing robust research practices in your project and/or research environment may no longer be a top priority. That’s OK. Focus on what is most important to you. You may be able to restart or intensify your efforts when your circumstances change.

## Rule 8: Share best practices and lessons learned

You can be proud of the practices that you have implemented to increase the robustness of your research. If you have the time and resources, look for opportunities to support others in your research environment who would like to implement some of the practices that you have initiated. Consider how to disseminate these practices within your research group. Some strategies include writing and field-testing good documentation, making SOPs, working with other team members to implement new strategies, or integrating training into the onboarding process for new team members. Document the steps that you took to make your work more robust in a repository, so in the future you can recycle your previous work as you move to new projects or change research groups. Provide advice and support to help others improve their own practices. Learning by doing is a great way to convince people to work differently [17]. Junior group members may look to you for guidance, so have a conversation with them about robust practices even if other lab members are not convinced. Be a role model for junior and senior colleagues.

If you have the capacity and resources, you may choose to look for opportunities to amplify your efforts beyond your research group. Speak to colleagues in your department about practices that you have implemented. If there is no formal community in your department, consider setting one up. For example, you might start a journal club that focuses on robust research [10]. Make sure that the community is welcoming and open to new members [18–20].

While you may have learned about robust research in a formal course setting, there are other ways in which you can share your skills. For example, you might be able to incorporate some robust research practices into lab practical sessions. As a thesis advisor, you could suggest that undergraduate or graduate thesis work could incorporate robust research practices.

## Rule 9: Get credit and make your contributions visible

Document the work you do on robust research so that others can see that you are active in this area. You can do this on your own website or on repositories like Zenodo (https://zenodo.org/) or the Open Science Framework (OSF; https://osf.io/). Also, include this information on your Curriculum Vitae (CV). You might add sections listing datasets and protocols deposited in public repositories, or report other science improvement activities, such as training and community-building. Some funders now ask for evidence of working robustly, so this documentation can help you with future grant and fellowship applications.

You may also seek out recognition, networking, or career development opportunities for your work on robust practices by applying for awards, internal or external ambassador programmes, and grants or fellowships. If you are interested and have time, you can also consider getting involved in science improvement activities organized by your community, such as running training courses or lobbying for institutional change. These sorts of initiatives can help connect you to larger movements in science. Some researchers enjoy participating in or leading activities to promote organizational change [11].

## Rule 10: Seek out supportive future employers who value your skills and interests

Many participants in robust research workshops are ECRs, who tend to change jobs every few years. If you are changing institutions or organizations, make sure your next job is in a team that shares your values, including (but certainly not limited to) robust research. Look for opportunities to work with people who value your skills and will support you in your efforts [21,22].

Start by defining your values. Think about what matters most to you. This includes your career and the way you want to do research but also your work–life balance and the type of environment in which you want to work. Next, spend time researching the people who you want to work with, as well as the institutions or organizations where they are located.

Check the research team’s and institution’s/organization’s track record online. What are their stated values, priorities, or philosophies? Do they use robust research practices in their research? Do they have clear policies on training, support, and promotion that are aligned with their and your values? In an interview, ask the team leader about their priorities and philosophies. Importantly, also speak with current and former team members. You can ask them about research practices, but also about the team leader. Do team members have freedom to explore their own interests? Is the team leader open to feedback from team members? Do team members get useful feedback from their team leader and support when they need it?

Finally, consider career trajectories that can incorporate work in robust research or science improvement. This could include meta-research positions or research support roles such as data stewards, research software engineers, librarians, public engagement officers, community managers, and educators in robust research. Also, consider positions in organizations that focus on changing research practices (e.g., infrastructure developers, innovative publishers, funders). Fields like these are all critical for the day-to-day practice of robust research, and opportunities will likely expand in coming years.

## Limitations

These “Ten Simple Rules” are based on the experiences of past participants in two robust research courses and have several limitations. While individual “rules” have been tested by course participants, it is unclear whether the whole set of rules is effective in implementing robust research practices. We welcome feedback from readers on the effectiveness of this set of rules. A number of factors might affect generalizability. First, participants and authors came mostly from a few research fields, including neuroscience, biology, psychology, and health research, and predominantly worked in academic research environments. Researchers in other research areas or environments may need different approaches to implement robust research practices. Furthermore, the tips were based on authors’ experiences in environments where junior researchers are able to question existing practices. Power structures and research cultures in some environments may prevent researchers, especially those early in their careers, from advocating for or implementing new practices. Finally, while the tips and hints provided in this article are targeted towards individuals, it is important to acknowledge that implementing robust research practices on a broad scale will require systemic changes. This includes adjusting incentives, rewards, and working conditions to encourage robust research practices.

## Conclusions

Training in robust research practices is an enriching and rewarding aspect of scientific education. However, participants may experience challenges when attempting to apply what they have learned in the classroom to their own research projects and in their wider research environment. Our “Ten Simple Rules” provide participant-informed guidelines that can help anyone make the most of their robust research training. Implementation of new research practices requires (1) prioritizing and planning which practices to implement, which includes obtaining support and dealing with the social challenges of convincing others involved in your research project of the added value of new practices; (2) managing problems that arise during implementation; and (3) making reproducible research and open science practices an integral part of your future research career. The guidelines presented here can help researchers to address these challenges. We hope that acceptance of robust research practices will continue to grow in the future, which will facilitate their implementation.

## Supporting information

S1 Text Methodological details of the virtual brainstorming event. While the virtual brainstorming format has been described previously [5,6], this Supporting information provides additional details about the specific event that led to this paper.
(DOCX)

S2 Text Tips for course organizers. The Supporting information outlines actions that course organizers can take to prepare and support course participants in implementing what they have learned once they return to their research environment.
(DOCX)
