# Supplementary material for: Ten simple rules for implementing open and reproducible research practices after attending a training course
Source: PLoS Comput Biol. 2023 Jan 5;19(1):e1010750. doi: 10.1371/journal.pcbi.1010750 (PMC9815586; doi:10.1371/journal.pcbi.1010750)
Supplement: S1 Text — While the virtual brainstorming format has been described previously [5,6], this Supporting information provides additional details about the specific event that led to this paper. (DOCX) [file pcbi.1010750.s001.docx]

S1 Text for **Ten simple rules for implementing open and reproducible research practices after attending a training course**

**Methodological details for the virtual brainstorming event**

The event organizers (VH, CH, HL, EML, TLW) invited participants of previous training courses to attend a virtual brainstorming event in September 2021 to discuss what happens “When education meets reality”, i.e. how training in robust research practices is put into practice. The virtual brainstorming consisted of (1) a virtual networking event, (2) an asynchronous virtual brainstorming, and (3) a virtual open space, where participants could join or lead in-depth discussions (format based on the event described here <https://wellcomeopenresearch.org/articles/6-156>).

The event was held with organizers and 27 participants over three days with discussions each day focusing on slightly different topics. The following questions that were discussed on the second and third day during the virtual in-person discussions and written asynchronous virtual brainstorming on the platform MS Teams formed the basis for the tips presented in this paper.

1. Which practices that you learned about in the course did you manage to implement in your day-to-day work? How easy was it to implement practices? Were there things that just weren't feasible for you?
2. Were there Open Science/ reproducibility practices that you already engaged in before the course and simply continued?
3. What things made it challenging for you to implement what you learned in the course? Have you encountered resistance? Are there strategies that helped you to overcome or mitigate these barriers?
4. Reproducibility hacks. What tricks did you learn that made implementation easier?
5. What do you wish you had known sooner?
6. How has your motivation to implement practices changed since the course? How do you stay motivated?
7. What can course organizers do to help you with implementing practices? What do you wish that you'd learned about implementation during the course?
8. Has the course affected your career trajectory in any way?
9. Did you try to make changes beyond your own practice, e.g. in your lab, institute, university?
10. Which stakeholders (beyond your research group) would you like to be included in conversations on implementing robust research practices?

No formal procedure was used to determine the rules. Event organizers discussed participants’ responses describing their experiences, and distilled this information into a set of rules. An outline and several drafts were shared with course participants to solicit feedback and the manuscript was adjusted based on participants’ comments.
