# Supplementary material for: Ten simple rules for implementing open and reproducible research practices after attending a training course
Source: PLoS Comput Biol. 2023 Jan 5;19(1):e1010750. doi: 10.1371/journal.pcbi.1010750 (PMC9815586; doi:10.1371/journal.pcbi.1010750)
Supplement: S2 Text — The Supporting information outlines actions that course organizers can take to prepare and support course participants in implementing what they have learned once they return to their research environment. (DOCX) [file pcbi.1010750.s002.docx]

S2 Text for **Ten simple rules for implementing open and reproducible research practices after attending a training course**

**Tips for Course Organizers**

Organizers can play a critical role in preparing participants for the challenges that they may encounter when attempting to implement new robust research practices in their research group. Past course participants emphasized several actions that organizers can take.

**When designing a course…**

1. **Consider participants’ background and research type when designing materials.** As you design your course, think about how implementation might differ for researchers from different research fields or countries. Consider the level of detail that a new graduate student might need to implement the practices discussed, compared to an experienced postdoc or an independent investigator. Also, consider how implementation might differ for someone who is designing their study, compared to someone who is collecting data, analyzing data or writing their paper. Providing clear information about what audience the course is designed for will help participants to determine whether the course is a good fit for their situation and skills. Asking for information about your participants when they register will help you to tailor the content to their needs.
2. **Cover a range of topics in your course.** Different topics resonate with different participants, and allow people to select practices that are more suited to their project, skills and research environment. Partnering with other lecturers or tutors who have expertise in different areas can be an efficient way of providing high quality content, without investing extensive time in learning many new skills.
3. **Talk about top-down robust research policies.** If you have a homogenous audience, share institutional policies or funder policies that are relevant to the course content (e.g. open data or open access publishing policies). These are important tools which equip participants to advocate for change when speaking with researchers in leadership positions. If participants come from different institutions or fields, give participants time to learn about their own institutions’ or funders’ policies related to course topics. Help participants to identify local resources, such as librarians or statisticians, that support implementation of robust research practices.
4. **Include information on “soft skills” related to behavioral change in research groups.** Course participants will often need to convince others before they can implement changes. Share techniques and resources to make this easier. For example, you may consider a session on “How to have a conversation with your colleagues”, which explores common concerns that researchers may have about implementing specific practices and presents strategies and materials for addressing these concerns. Alternatively, you might arrange a panel where past participants talk about challenges and successes that they experienced when attempting to implement practices from the course into their own research.
5. **Give participants time to start implementing practices.** Include time in your workshop for participants to start applying basic skills they have learned to their own research. Instructors can also help participants to overcome early roadblocks. Getting started during the workshop also makes it easier to continue later.
6. **Consider offering a series of sessions, instead of covering many topics in a short period of time:** Single or multi-day courses that cover many topics can leave participants feeling overwhelmed. Attendees may struggle to determine what they can and should implement. They might also be expected to resume normal research activities immediately after the workshop, making it difficult to find time to implement new practices. Instructors may no longer be accessible if they encounter obstacles. An alternative to this “buffet style'' approach is to spread sessions out over time, allowing participants to implement one practice before learning about the next. Questions that arise while implementing the practice discussed in the previous sessions can be addressed at the beginning of the next session. This also gives participants an opportunity to share how they implemented a practice in their own research, providing valuable opportunities for group learning and problem solving.

**When participants return to their own research environments…**

1. **Make your teaching materials and resources open and re-usable.** Course participants can use these materials to prepare talks for lab meetings and meetings with colleagues.
2. **Create (and support) communities.** Set up a way for course alumni to stay in touch with course organizers and each other. During the course, include information on how to find a robust research community, and how to start a new community if existing communities are not accessible or do not meet participants' needs.
3. **Support implementation.** Consider organizing implementation days or weeks for participants to focus on implementing specific skills and sharing problems and solutions. Use an online discussion platform or virtual meeting space to facilitate discussions.
4. **Consider follow-up events.** Be available to do workshops in lab meetings or departmental meetings, if possible, and give course participants materials and resources to organize training events
